# Supplementary material for: Multi‐dataset identification of innovative feature genes and molecular mechanisms in keratoconus
Source: J Cell Mol Med. 2024 Sep 19;28(18):e70079. doi: 10.1111/jcmm.70079 (PMC11412914; doi:10.1111/jcmm.70079)
Supplement: Supplementary file 2 — Material S2. [file JCMM-28-e70079-s002.zip › 7.GSEA analysis/6.SNORA74B/KEGG_CHRONIC_MYELOID_LEUKEMIA.html]

Details for gene set KEGG\_CHRONIC\_MYELOID\_LEUKEMIA[GSEA]

|  || Dataset | SNORA74B.group.cls#h\_versus\_l |
| Phenotype | group.cls#h\_versus\_l |
| Upregulated in class | l |
| GeneSet | KEGG\_CHRONIC\_MYELOID\_LEUKEMIA |
| Enrichment Score (ES) | -0.37596947 |
| Normalized Enrichment Score (NES) | -1.4771255 |
| Nominal p-value | 0.08086785 |
| FDR q-value | 0.348467 |
| FWER p-Value | 0.815 |
Table: GSEA Results Summary

  

Fig 1: Enrichment plot: KEGG\_CHRONIC\_MYELOID\_LEUKEMIA      
 Profile of the Running ES Score & Positions of GeneSet Members on the Rank Ordered List

  

| SYMBOL | TITLE | RANK IN GENE LIST | RANK METRIC SCORE | RUNNING ES | CORE ENRICHMENT || 1 | PIK3R5 | na | 170 | 0.800 | 0.0796 | No |
| 2 | CBLB | na | 5386 | 0.212 | -0.0484 | No |
| 3 | SOS1 | na | 5971 | 0.193 | -0.0449 | No |
| 4 | E2F2 | na | 7913 | 0.138 | -0.0863 | No |
| 5 | BRAF | na | 8089 | 0.134 | -0.0772 | No |
| 6 | PIK3R1 | na | 8116 | 0.133 | -0.0640 | No |
| 7 | IKBKB | na | 8763 | 0.119 | -0.0700 | No |
| 8 | AKT2 | na | 9380 | 0.105 | -0.0767 | No |
| 9 | TGFBR2 | na | 9513 | 0.102 | -0.0697 | No |
| 10 | KRAS | na | 9766 | 0.097 | -0.0667 | No |
| 11 | MECOM | na | 10361 | 0.086 | -0.0748 | No |
| 12 | CBL | na | 11198 | 0.072 | -0.0913 | No |
| 13 | MDM2 | na | 11211 | 0.071 | -0.0841 | No |
| 14 | CTBP1 | na | 11288 | 0.070 | -0.0789 | No |
| 15 | TGFB2 | na | 11803 | 0.063 | -0.0871 | No |
| 16 | PIK3R2 | na | 12946 | 0.047 | -0.1151 | No |
| 17 | PIK3CA | na | 13079 | 0.046 | -0.1140 | No |
| 18 | SMAD4 | na | 13214 | 0.045 | -0.1132 | No |
| 19 | CTBP2 | na | 13433 | 0.042 | -0.1150 | No |
| 20 | RB1 | na | 13606 | 0.040 | -0.1157 | No |
| 21 | SOS2 | na | 13685 | 0.039 | -0.1138 | No |
| 22 | PTPN11 | na | 13916 | 0.037 | -0.1165 | No |
| 23 | CBLC | na | 13962 | 0.036 | -0.1140 | No |
| 24 | MAP2K2 | na | 14904 | 0.025 | -0.1385 | No |
| 25 | STAT5B | na | 15213 | 0.022 | -0.1450 | No |
| 26 | CCND1 | na | 15223 | 0.022 | -0.1429 | No |
| 27 | PIK3CB | na | 15769 | 0.017 | -0.1568 | No |
| 28 | BCR | na | 16207 | 0.013 | -0.1681 | No |
| 29 | SMAD3 | na | 16318 | 0.011 | -0.1701 | No |
| 30 | CHUK | na | 16402 | 0.010 | -0.1714 | No |
| 31 | MAPK3 | na | 16753 | 0.007 | -0.1808 | No |
| 32 | HDAC1 | na | 16993 | 0.005 | -0.1872 | No |
| 33 | E2F3 | na | 17092 | 0.004 | -0.1896 | No |
| 34 | AKT1 | na | 18366 | -0.010 | -0.2252 | No |
| 35 | PIK3CG | na | 18884 | -0.016 | -0.2385 | No |
| 36 | BAD | na | 19265 | -0.021 | -0.2473 | No |
| 37 | TP53 | na | 19572 | -0.024 | -0.2536 | No |
| 38 | HRAS | na | 19699 | -0.025 | -0.2546 | No |
| 39 | CDKN2A | na | 19773 | -0.026 | -0.2539 | No |
| 40 | CDKN1B | na | 20194 | -0.030 | -0.2628 | No |
| 41 | SHC3 | na | 20230 | -0.031 | -0.2606 | No |
| 42 | PIK3CD | na | 20354 | -0.032 | -0.2607 | No |
| 43 | MAPK1 | na | 20606 | -0.035 | -0.2642 | No |
| 44 | IKBKG | na | 20820 | -0.038 | -0.2664 | No |
| 45 | RAF1 | na | 21074 | -0.041 | -0.2694 | No |
| 46 | HDAC2 | na | 22146 | -0.053 | -0.2946 | No |
| 47 | SHC1 | na | 22752 | -0.062 | -0.3056 | No |
| 48 | ABL1 | na | 23665 | -0.076 | -0.3239 | No |
| 49 | CDK4 | na | 24176 | -0.084 | -0.3298 | No |
| 50 | GRB2 | na | 24997 | -0.098 | -0.3431 | No |
| 51 | MYC | na | 25534 | -0.108 | -0.3471 | No |
| 52 | NFKB1 | na | 26534 | -0.128 | -0.3624 | Yes |
| 53 | CRK | na | 26740 | -0.134 | -0.3542 | Yes |
| 54 | TGFBR1 | na | 27137 | -0.142 | -0.3506 | Yes |
| 55 | E2F1 | na | 27392 | -0.149 | -0.3422 | Yes |
| 56 | CDK6 | na | 27632 | -0.155 | -0.3327 | Yes |
| 57 | ARAF | na | 27916 | -0.162 | -0.3238 | Yes |
| 58 | NRAS | na | 27969 | -0.164 | -0.3080 | Yes |
| 59 | NFKBIA | na | 28570 | -0.182 | -0.3061 | Yes |
| 60 | STAT5A | na | 28673 | -0.185 | -0.2895 | Yes |
| 61 | BCL2L1 | na | 28814 | -0.189 | -0.2736 | Yes |
| 62 | CRKL | na | 29271 | -0.203 | -0.2653 | Yes |
| 63 | PIK3R3 | na | 29453 | -0.208 | -0.2486 | Yes |
| 64 | RELA | na | 29651 | -0.214 | -0.2316 | Yes |
| 65 | TGFB1 | na | 30144 | -0.231 | -0.2214 | Yes |
| 66 | RUNX1 | na | 31103 | -0.273 | -0.2202 | Yes |
| 67 | MAP2K1 | na | 31129 | -0.275 | -0.1919 | Yes |
| 68 | CDKN1A | na | 31225 | -0.280 | -0.1651 | Yes |
| 69 | TGFB3 | na | 31437 | -0.291 | -0.1405 | Yes |
| 70 | GAB2 | na | 32350 | -0.344 | -0.1305 | Yes |
| 71 | SHC4 | na | 32485 | -0.353 | -0.0971 | Yes |
| 72 | SHC2 | na | 34407 | -0.652 | -0.0837 | Yes |
| 73 | AKT3 | na | 34657 | -0.880 | 0.0020 | Yes |
Table: GSEA details [plain text format]

  

Fig 2: KEGG\_CHRONIC\_MYELOID\_LEUKEMIA      
 Blue-Pink O' Gram in the Space of the Analyzed GeneSet

  

Fig 3: KEGG\_CHRONIC\_MYELOID\_LEUKEMIA: Random ES distribution      
 Gene set null distribution of ES for **KEGG\_CHRONIC\_MYELOID\_LEUKEMIA**
